# Fractures in the Setting of Constriction Band Syndrome: A Case Series of Three Patients with Extremity Fractures at the Site of a Constriction Band

**DOI:** 10.3390/children9060876

**Published:** 2022-06-12

**Authors:** Yerin Cho, Leeann Qubain, Melissa Esparza, Judson Karlen, Timothy Schaub, Mohan V Belthur

**Affiliations:** 1 University of Arizona College of Medicine—Phoenix, Phoenix, AZ 85004, USA; yerinahur@email.arizona.edu; 2 Orthopedic Surgery, Phoenix Children’s Hospital, Phoenix, AZ 85016, USA; melissa.esparza@gmail.com (M.E.); jkarlen@phoenixchildens.com (J.K.); mbelthur@phoenixchildrens.com (M.V.B.); 3 Plastic Surgery, Phoenix Children’s Hospital, Phoenix, AZ 85016, USA; tschuab@phoenixchildrens.com

**Keywords:** congenital, fracture, constriction band syndrome, intramedullary stabilization, k-wire, case series

## Abstract

Constriction band syndrome (CBS) is a rare condition where fibrous bands constrict one or more parts of the fetus with varying manifestations such as autoamputation, acrosyndactyly, and neuropathy. However, isolated extremity fractures in the setting of constriction band syndrome are even more rare, with only two reported cases in the literature. There are few guidelines on the management of CBS due to small case numbers, the variability of presentation between patients, and the lack of consensus on etiology and pathogenesis. In this small case series, we describe the presentation and management of three patients at our institution with extremity fractures at the site of severe constriction bands with neurologic injuries or vascular compromise. We also review the literature on this topic to provide further context. Intramedullary stabilization of the fracture with a k-wire allowed for soft tissue healing in two of our patients.

## 1. Introduction

Constriction band syndrome (CBS) is a rare condition in which fibrous amniotic bands entangle one or more parts of the fetus leading to various congenital anomalies. It has an estimated incidence of one in 1200 to one in 15,000 live births [1,2] with equal expression in males and females. There is currently no firm consensus on the etiology of CBS; however, there are two theories often used in the literature. The intrinsic theory, first described by Streeter in 1930, suggests that the amniotic band is formed from the embryo failing to develop properly [3]. In contrast, the extrinsic theory proposes that an early rupture of the amniotic sac leads to the creation of fibrous amniotic bands, as described by Torpin in 1965 [4]. A lack of firm consensus on its etiology coupled with a heterogenic clinical presentation may explain the many names of this syndrome, such as congenital constriction ring syndrome (CCRS) and amniotic band syndrome (ABS) [2].

Amniotic bands most commonly affect the extremities, with a predilection for distal digits [2]. Manifestations of CBS include acrosyndactyly, neurovascular compromise, neuropathy, lymphedema, congenital amputations, and other deformities of the hands and feet [5,6,7]. Although less common, it can also present with multiple craniofacial and truncal abnormalities [6].

Fractures in the setting of constriction band syndrome are exceedingly rare. Small case series and case reports of pseudoarthrosis have been reported in the literature [8,9,10,11,12,13,14,15,16,17]. While fractures may be considered on the same spectrum of pseudarthrosis and other osseous defects, the chronicity and natural history of more acute fractures is distinct from true pseudarthroses. There are only two reports in the literature of isolated extremity fractures in the setting of a congenital constriction band (CCB) [18,19]. Here, we report a small case series on the presentation and management of three patients at our institution who were identified to have an upper or lower extremity fracture at the site of a CCB shortly after birth. Institutional Review Board approval was obtained to perform this retrospective review. Demographic data, medical course, treatment details, and clinical outcomes were investigated. A literature review was performed to identify other reported cases. The purpose of this study is to review the literature on extremity fractures seen in CBS and describe the presentation, management, and short-term outcome of patients treated at our institution for extremity fractures in the setting of CBS.

## 2. Case Presentation

**Figure 1:**
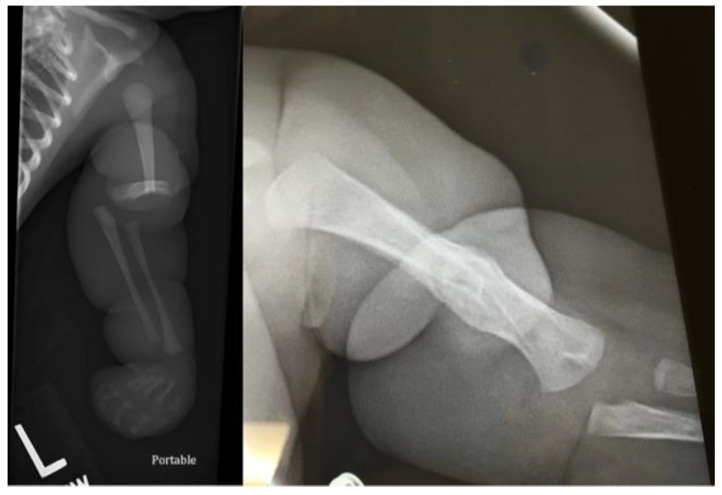
Radiographs of Patient 1: preoperative and 1-year postoperative with healed fracture (left to right).

**Figure 2:**
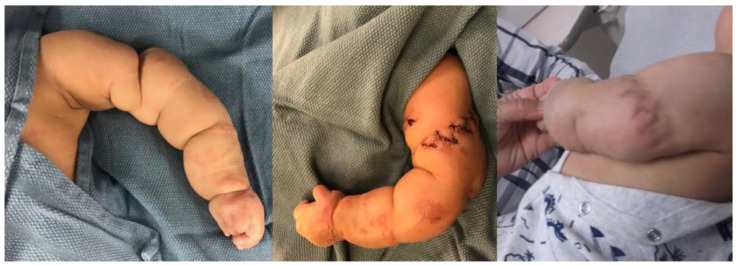
Clinical photos of Patient 1: preoperative, intraoperative, and 1-year postoperative (left to right).

Patient 1 was born at 38 weeks gestational age with a left distal humeral shaft fracture at the site of a CCB. On initial examination by the plastic surgery team, there was severe banding with a tight sac encompassing the hand and band extending to the upper arm. A sterile bedside procedure was performed to remove the sac from the hand. The hand was completely flaccid with no noted function of the radial, median, or ulnar nerves that could be elicited. However, there did appear to be good perfusion of the hand, and ultrasound of the extremity confirmed appropriate flow of the brachial artery distal to the fracture. The humerus fracture was initially angulated 90 degrees, which was grossly realigned and immobilized (Figure 1). After the fracture had healed, the patient underwent delayed CCB release with multiple z-plasties and neurolysis of the median, ulnar, and radial nerves at 14 weeks of age by plastic surgery (Figure 2).

**Figure 3:**
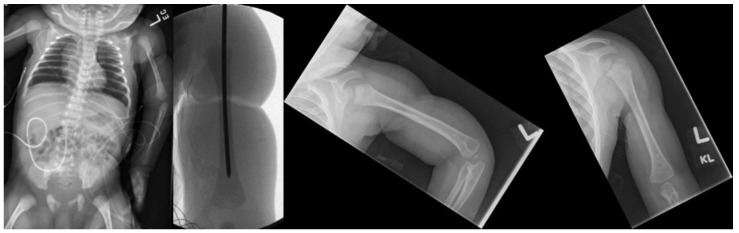
Radiographs of Patient 2: preoperative AP view, intraoperative AP view, and 1-year postoperative AP views of flexed and extended left upper extremity (left to right).

Patient 2 was born at 36 weeks gestational age with CCBs affecting her right lower and bilateral upper extremities. The left upper extremity CCB was severe, causing an open wound 270 degrees around the arm and a resultant humeral shaft fracture. She also had congenital amputations of the left index and ring fingers. Her arm was flaccid distal to the band and operative exploration demonstrated transection of the radial, median, and ulnar nerves and of the triceps and short head of the biceps. She underwent band release and repair of the median, ulnar, radial, and medial antebrachial cutaneous nerves by plastic surgery at 2 days of age. In order to facilitate the nerve exploration and repair and provide a stable environment to protect the repair, intramedullary k-wire fixation of the humerus fracture was performed by orthopedic surgery prior to the soft tissue procedures (Figure 3). At 2-week follow up, there was already substantial callus formation at the fracture site and the k-wire was removed in clinic. At 2-month follow up, the humerus fracture was fully healed. The patient gained some function of her left upper extremity with the ability to hold objects in her hand and demonstrate lateral pincer grasp, though her grip strength remained weak, causing limited function of her left hand.

**Figure 4:**
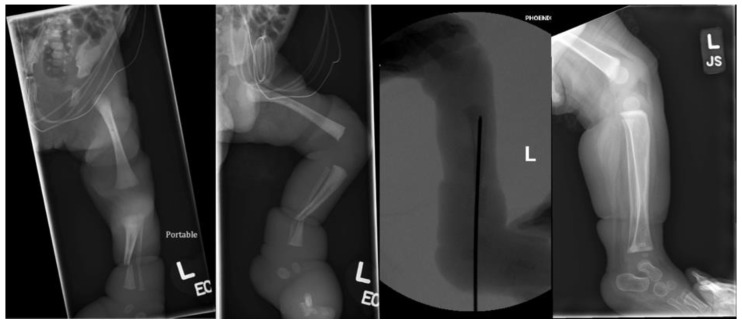
Radiographs of Patient 3: preoperative AP view, preoperative lateral view, intraoperative lateral view, and 1-year postoperative lateral view (left to right).

**Figure 5:**
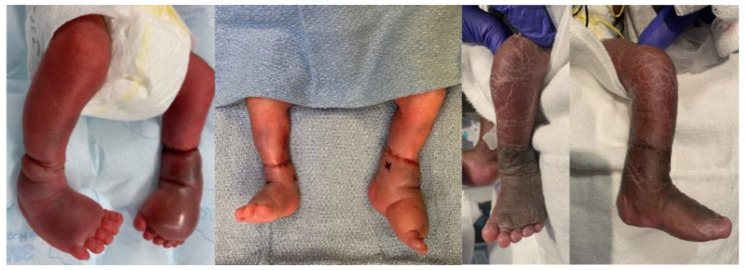
Clinical photos of Patient 3: preoperative, intraoperative, and 1-year postoperative.

Patient 3 was born premature at 28 weeks with CCBs involving the bilateral distal legs. On the left side, he was noted to have a deep circumferential supramalleolar band causing severe venous congestion and edema distal to the band with purple discoloration, sluggish capillary refill, and decreased pulses. Plastic surgery was initially consulted and took the patient to the OR for urgent constriction band release due to concern for limb ischemia. Orthopedics was also consulted due to distal third tibia and fibula shaft fractures at the site of the left CCB. After band release by plastic surgery, he was placed in a long leg splint on the left side. However, venous congestion began to worsen again over the following days and the plastic surgery team requested further stabilization of the fracture to protect the soft tissue repair. The patient returned to the OR on day of life 2 and the orthopedics team performed percutaneous retrograde intramedullary fixation of the tibia fracture with a 1.6 mm k-wire (Figure 4). The k-wire and splint were removed after 3 weeks, with good soft tissue healing, excellent fracture alignment, and abundant callus formation at that time. At 2-month follow up, the fracture and soft tissues were fully healed, with normal neurovascular exam. At 11-month follow up, the patient continued to do well and was pulling to a stand and walking with assistance without any orthotic use (Figure 5).

## 3. Discussion

Constriction band syndrome is a highly variable spectrum of congenital abnormalities that occur in the presence of an amniotic band. Due to its widely heterogenetic clinical presentation, there is no set of consistent findings required for diagnosis. Rather, CBS is associated with the presence of constrictive rings, limb defects, neural defects, and craniofacial abnormalities [6,7,20]. The amniotic band most commonly affects the upper or lower extremities, with a predilection for distal limbs and digits [9,11,12,13,14,15,17]. The degree of banding can range from superficial indentations to deep compressions that extend into the bone, nerves, and vascular supply [5,21]. Vascular compromise distal to the band may cause severe vascular, lymphatic, and/or neural damage. Resulting ischemia may also lead to osteomyelitis and even partial or complete amputations [22].

This variability in clinical presentations thus requires management to be individualized in each case. Shallow bands that do not present with vascular or neurological compromise may not require any surgical intervention aside from cosmetic repair. On the other hand, deep rings associated with neurovascular compromise may require emergency limb-sparing band release [22,23]. Therefore, it is essential to monitor the affected limb to assess any changes in circulatory status [10,13,17].

In our review of the literature, pseudoarthrosis of the tibia and fibula was the most common osseous defect in CBS [11,12,13,14,15,17]. In a case series of seven patients with tibial pseudarthrosis associated with CBS, Naik et al. [24] differentiated the patients in their series with hypertrophic nonunions and angular deformity at the pseudarthrosis site compared to fractures or tibial bowing. In comparison to the relatively more common occurrence of pseudarthrosis, we have found only two cases of recent fracture caused by an amniotic band.

Masmoudi et al. published a case report of an amniotic band causing fracture of the tibia and fibula. Their patient had no signs of vascular compromise, with no skin breaches and normal capillary pulses and refill time. Non-operative treatment was reported to be successful [19].

A fracture of the forearm in an infant due to an amniotic band was reported by Angelis et al. Severe swelling, neural damage, and vascular compromise were noted. Band release was performed on day 4 and the fracture was reduced and immobilized. Circulation improved but the patient deceased on day 16 due to vascular distress [18].

Ho et al. report a similar course of management in a rare case of pseudoarthrosis of the forearm. In contrast to Angelis et al., initial presentation of the limb was viable without any evidence of vascular compromise. Severe edema, cyanosis, and diminished pulses were present only after fluid resuscitation. Band release was performed on day 2 and the forearm was immobilized and healed with no further surgical intervention [10].

The management of patients with CBS has evolved over time and fractures in the setting of a congenital constriction band are rare. These patients often require a multidisciplinary approach between plastic surgery and orthopedic surgery. Future studies could improve upon the limitations of our small sample size and limited follow-up. However, this can be difficult in the setting of a rare condition such as CBS. To the best of our knowledge, there are only two previous case reports of fractures caused by an amniotic band in the literature. Here, we present a series of three patients with extremity fractures at the site of severe constriction bands with neurologic injuries or vascular compromise. As seen in two of these patients, intramedullary stabilization of the fracture with a k-wire may be indicated in some cases to provide bony stability, reduce tension on the soft tissue repair and allow for soft tissue healing.

## Data Availability

Due to the nature of this article as a case series, there are no external data available for readers to access. All clinical information was taken from chart review at Phoenix Children’s Hospital and cannot be released due to HIPAA regulations.
